# ADL-dependency, D-Dimers, LDH and absence of anticoagulation are independently associated with one-month mortality in older inpatients with Covid-19

**DOI:** 10.18632/aging.103583

**Published:** 2020-06-23

**Authors:** Guilhem Bousquet, Géraldine Falgarone, David Deutsch, Sophie Derolez, Marilucy Lopez-Sublet, François-Xavier Goudot, Khadaoudj Amari, Yurdagul Uzunhan, Olivier Bouchaud, Frédéric Pamoukdjian

**Affiliations:** 1 AP-HP Hôpital Avicenne, Oncologie Médicale, Bobigny 93000, France; 2 Université Sorbonne Paris Nord, INSERM, U942, Cardiovascular Markers in Stressed Conditions, MASCOT, Bobigny 93000, France; 3 AP-HP Hôpital Avicenne, Unité de Médecine Ambulatoire (UMA), Bobigny 93000, France; 4 Université Sorbonne Paris Nord, Villetaneuse 93430, France; 5 AP-HP Hôpital Avicenne, Gastroentérologie et Oncologie Digestive, Bobigny 93000, France; 6 AP-HP Hôpital Avicenne, Rhumatologie, Bobigny 93000, France; 7 AP-HP Hôpital Avicenne, Médecine Interne, ESH Hypertension Excellence Centre, Bobigny 93000, France; 8 AP-HP Hôpital Avicenne, Cardiologie, Bobigny 93000, France; 9 APHP Hôpital Avicenne, Service de Médecine Gériatrique, Bobigny 93000, France; 10 AP-HP Hôpital Avicenne, Pneumologie, Bobigny 93000, France; 11 Université Sorbonne Paris Nord, INSERM, U1272, Hypoxia and Lung, Bobigny 93000, France; 12 AP-HP Hôpital Avicenne, Infectious Diseases, Bobigny 93000, France; 13 EA 3412, Laboratoire Educations et Pratiques de Santé, Bobigny 93000, France

**Keywords:** COVID-19, geriatric assessment, mortality, predictive biomarkers, anticoagulation

## Abstract

Background: To assess factors associated with one-month mortality among older inpatients with Covid-19.

Results: The mean age was 78 ± 7.8 years, 55.5% were men, CT scan lung damage was observed in 76% of the patients (mild 23%, moderate 38%, extensive 22%, and severe 7%). The mortality rate was 26%. Dependency/Activities of Daily Living (ADL) score ≤ 5/6, D-Dimers, LDH, and no anticoagulation by reference for curative were independently associated with one-month mortality. A score derived from the multivariate model showed good calibration and very good discrimination (Harrell’s C index [95%CI] = 0.83 [0.79-0.87]).

Conclusion: ADL-dependency, high serum levels of D-Dimers and LDH and the absence of anticoagulation were independently associated with one-month mortality among older inpatients with Covid-19.

Methods: 108 consecutive older inpatients aged 65 and over with Covid-19 confirmed by RT-PCR and/or typical CT chest scan were prospectively included in a French single-centre cohort study from March to April 2020. A systematic geriatric assessment was performed. Covariates were lymphocyte count, serum levels of albumin, C-Reactive Protein, D-Dimers and Lactate Dehydrogenase (LDH), anticoagulation level, and exposure to the hydroxychloroquine and azithromycin combined therapy. Cox uni- and multivariate proportional-hazard regressions were performed to identify predictors of one-month mortality.

## INTRODUCTION

Since the end of 2019, the SARS-Cov-2 pandemic (named Covid-19) exposes older patients to the risk of early death [1–3]. As with other diseases, chronological age should not be the only element in the therapeutic decision.

To date, factors associated with short-term mortality among older inpatients with Covid-19 have not been characterized. Given the heterogeneity of the older inpatient population, these factors are needed to avoid under- and over-treatment, particularly intensive care.

We used the Geriatric Assessment (GA) to try to identify predictive factors associated with one-month mortality among older inpatients with Covid-19 [4].

## RESULTS

### Patients

The Covid-19 outbreak has been particularly severe in Paris and its suburbs since February 2020. The university hospital of Paris-Seine Saint Denis (Avicenne hospital) set aside nearly 200 hospital beds for Covid-19 patients. In this health emergency, a Geriatric Assessment (GA) was systematically performed for older inpatients with Covid-19 to help clinical teams in their therapeutic strategy. This prospective observational cohort study consecutively included all older (65 and over) inpatients with a Covid-19 diagnosis. The diagnosis of Covid-19 was based on a positive SARS-Cov-2 RT-PCR test on a nasopharyngeal sample [5] and/or on a typical CT chest scan [6]. Informed consent was obtained from the patients before inclusion in accordance to national ethical rules.

Three hundred and twenty-five new consecutively admitted patients for a confirmed Covid-19 infection were recorded between 03/28/2020 and 04/13/2020, of whom 120 (37%) concerned individuals 65 years of age or older. We assessed 108 (90%) of them.

### Baseline characteristics of the patients

Among the 108 Covid-19 patients studied, RT-PCR testing for SARS-Cov-2 was positive for 85% of the patients (n=92/108), and CT scan was available for 84% (n=91/108). On CT scans, there was no lung disease for 10% (n=9/91), mild damage for 23% (n=21/91), moderate damage for 38% (n=35/91), extensive damage for 22% (n=20/91), and severe damage for 7% (n=6/91).

The mean age was 78.4 ± 7.8 years (min-max: 66-95), and 55.5% were men. The geriatric domains impaired concerned ranged from 16% (BMI < 21 kg/m^2^) to 87% (muscle weakness). Median serum levels of D-Dimers and LDH were 1308.5 ng/mL and 341.5 UI/L respectively. 93/108 patients had an anticoagulation either curative (30%) or preventive (56%). 27/108 of the patients received the combination of hydroxychloroquine and azithromycin for 1 to 9 days (Table 1).

**Table 1:** Baseline characteristics of 108 older inpatients with Covid-19, uni- and multivariate factors associated with one-month mortality.

**Variables**	**Whole cohort N = 108 (%)**	**Univariate analysis**		**Multivariate analysis**		
		**HR [95%CI]**	P*	**aHR [95%CI]**	P*	**Scoring**
Age (y), median (IQR)	78 (13)	1.05 [1.00-1.10]	**0.03**	-		
Gender (male)	60 (55.5)	1.45 [0.67-3.11]	0.34			
Comorbidities						
Total CIRSG ≥ 11	57 (53)	2.38 [1.01-5.62]	**0.04**	-		
Hypertension	77 (71)	1.34 [0.54-3.32]	0.52			
Diabetes	30 (28)	1.57 [0.72-3.44]	0.25	-		
Dependency						
ADL ≤ 5/6	54 (50)	6.65 [2.30-19.2]	**0.0004**	4.33 [1.39-13.5]	**0.01**	4
IADL ≤ ¾	68 (63)	7.93 [1.88-33.4]	**0.004**	-		
Nutrition						
BMI < 21 kg/m2	17 (16)	0.89 [0.34-2.35]	0.81			
Weight loss ≥ 5% (yes)	49 (45)	1.38 [0.65-2.93]	0.40			
Mobility						
Muscle weakness (yes)	94 (87)	4.92 [0.66-36.6]	0.12	4.44 [0.57-34.5]	0.15	4
Depressed mood						
Mini GDS ≥ 1/4	65 (60)	2.27 [0.95-5.41]	0.06	2.30 [0.81-6.49]	0.11	2
Covariates (median, IQR)						
Albumin level (g/L)	27 (7.0)	0.94 [0.87-1.02]	0.12	-		
CRP level (mg/L)	85.5 (110.5)	1.00 [0.99-1.01]	0.23	-		
Lymphocyte count	955 (650.0)	1.00 [0.99-1.00]	0.89			
D-dimers (ng/mL)	1308.5 (1405.0)	1.00 [1.00-1.01]	**0.02**	1.00 [1.00-1.00]	**0.0008**	1
LDH (IU/L)	341.5 (195.5)	1.00 [0.99-1.00]	0.08	1.00 [1.00-1.00]	**0.03**	1
Intensive cares (yes)	7 (6.5)	1.02 [0.30-3.47]	0.97			
Converting enzyme inhibitors (yes)	42 (39)	1.18 [0.55-2.52]	0.67			
Anticoagulation			0.12		**0.02**	
Curative	32 (30)	1 (reference)		1 (reference)		0
Preventive	61 (56)	1.45 [0.55-3.78]		1.20 [0.43-3.31]		1
None	15 (14)	2.91 [1.00-8.47]		4.20 [1.36-12.9]		4
Hydroxychloroquine + azithromycin (yes)	27 (25)	0.49 [0.19-1.29]	0.15	-		

* Log rank test; Bold: significant *P* value at the threshold of 5%; IQR: Inter Quartile Range; HR: Hazard Ratio; aHR: adjusted HR; Continuous variables are expressed by one IQR of more.

CIRSG: Cumulative Illness Rating Scale Geriatric; ADL: Activity of Daily Living; IADL: Instrumental-ADL; BMI: Body Mass Index; Mini-GDS: Mini Geriatric Depression Scale; CRP: C-Reactive Protein; LDH: Lactate Dehydrogenase.

### Univariate and multivariate factors associated with one-month inpatient mortality

All patients were followed up without loss until discharge from acute care unit. The median follow-up time was 10 days (IQR = 15) (min-max: 0-37). 7 patients (6.5%) were admitted to intensive care and three died. On 05/02/2020, the inpatient mortality rate was 26% (n=28/108).

In univariate analyses, age (per one IQR of more), comorbidities (total CIRSG ≥ 11), dependency (ADL ≤ 5/6 and IADL ≤ ¾), D-Dimers (per one IQR of more) were significantly associated with one-month inpatient mortality. None of the following were associated with one-month inpatient mortality: gender, CT chest scan damage, malnutrition (BMI < 21 kg/m^2^ or weight loss ≥ 5%), muscle weakness, depressed mood (mini GDS ≥ ¼), serum levels of albumin, age-adjusted D-Dimers, CRP and LDH, absolute lymphocyte cell count, anticoagulant therapy, and hydroxychloroquine and azithromycin combined therapy (Table 1).

In multivariate analyses, comorbidities (total CIRSG ≥ 11) were not anymore associated with mortality. Only ADL-dependency (aHR = 4.33 [1.39-13.5], *P* = 0.01), D-Dimers per one IQR of more (aHR = 1.00 [1.00-1.00], *P* = 0.0008), LDH per one IQR of more (aHR = 1.00 [1.00-1.00], *P* = 0.03), and no anticoagulation by reference for curative (aHR = 4.20 [1.36-12.9], *P* = 0.02) were significantly associated with one-month inpatient mortality (Table 1). There was no significant interaction between predictors (*P* for interaction ≥ 0.05).

### Derivation score for one-month inpatient mortality

**Figure 1:**
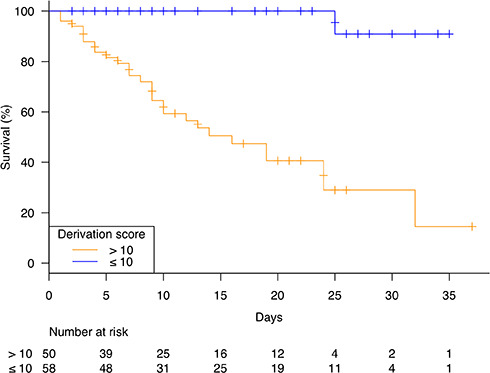
**Kaplan-Meier survival curves for short-term inpatients mortality according to derivation score.**

The derivation score ranged from 3 to 63 with a median score of 10 (IQR = 5). Two groups were identified: 58 patients (54%) were at low risk (3 to 10), and 50 (46%) at high risk (score > 10). Overall, the score was well calibrated (*P* = 0.24), and discrimination was very good with a Harrell’s C index of 0.83 (0.79-0.87). The Kaplan-Meier plot showed significant discrimination (*P* = 0.0004) across the two risk groups. In particular, the one-month inpatient risk of mortality was 9.1% (low risk), and 85.5% (high risk) respectively (Figure 1). For internal validation, using a bootstrapping method with 1000 resamples, the Harrell’s C index was 0.81 [0.75-0.88], close to the original C index. Overall, we showed that our prognostic score is reliable to predict short-term mortality in older inpatients with Covid-19.

## DISCUSSION

This is the first report on a prospective observational cohort study of older inpatients with Covid-19 that specifically assessed geriatric conditions and factors associated with one-month mortality. We found that ADL-dependency before hospitalization, serum levels of D-Dimers and LDH, and the absence of anticoagulation were the factors independently associated with one-month mortality in older inpatients with Covid-19.

To overcome the heterogeneity of older inpatients with Covid-19 in terms of comorbidities, dependency, nutrition, mobility and mood, we used the Geriatric Assessment to detect vulnerabilities. In this frail population where half of the patients had significant comorbidities and two-thirds had pre-admission dependency, the mortality rate of 26% is closed to the 34.5% mortality rate reported for 55 Chinese patients from Wuhan over 65 years [7]. In our study, we identified one clinical factor independently associated with one-month mortality: ADL-dependency (≤ 5/6) which is a typical complication of frailty among older adults [8]. We also identified two biological factors independently associated with one-month mortality, high serum levels of D-Dimers and LDH, previously reported as risk factors in younger patients [9, 10].

Strikingly, curative anticoagulation was strongly and independently associated with decreased risk of one-month mortality. Over-incidence of thromboembolism events in Covid-19 patients has been reported [11], and this protective effect of anticoagulation with high serum levels of D-Dimers suggest associated vascular impairment and possible direct effect of SARS-Cov-2 on normal endothelial cells [12].

From these four variables combined with depressed mood (i.e. mini-GDS) and muscle weakness, we derived a score to predict inpatient mortality with good calibration and very good discrimination. Other scores have been proposed to predict the risk of progression, but not for older inpatients [13]. This is a major strength of our study. Thus, for a patient over 65 years with pre-admission ADL-dependency, muscle weakness and depressed mood, and high serum levels of D-Dimers, the risk of short-term mortality is very high (85%), and should lead to cautious routing to intensive care. In contrast, a patient with no ADL-dependency and no depressed mood, and thus a very low risk of one-month mortality, should be actively transferred to intensive care unit if his/her respiratory condition requires it, regardless of his/her chronological age. Among older patients with Covid-19, as with most diseases [14], chronological age should not be the only factor considered for therapeutic decision, to avoid under- or over-treatment.

In addition, our original score includes thromboembolic-related risk of death and could help to choose the appropriate level of anticoagulation. Let us consider the real case of a 74-year-old woman hospitalized after 8 days of symptoms, with a muscle weakness, D-Dimers at 2950 ng/mL and LDH at 290 UI/L. Thus, the score is 8 (low risk). However, in the absence of anticoagulation, the score is 12 with a high risk of mortality. For this reason, she should be offered at least preventive anticoagulation.

The limitations of our study are the one-single center recruitment with the limited number of patients, and the absence of external validation. This is counterbalanced by a rigorous methodology and high prognostic performances of our scoring system to predict short-term mortality in older inpatients with Covid-19. Our results are also of particular importance in identifying the most at-risk older patients and protecting them as well as possible from the second wave, once confinement measures are lifted. In the latter case, a further validation of our study results will be required.

## CONCLUSIONS

ADL-dependency, high serum levels of D-Dimers and LDH and the absence of anticoagulation were independently associated with one-month mortality among older inpatients with Covid-19. A simple derivation score was developed to help clinicians in their daily therapeutic strategy.

## MATERIALS AND METHODS

### Demographic and disease characteristics

Demographic data (age, gender), and severity of the Covid-19 based on CT chest scan for lung damage extent (none 0%, mild < 10%, moderate 10-25%, extensive 25-50%, or severe > 50%) were collected at the first GA [6].

### The geriatric assessment (GA)

The GA was performed by two clinicians (GB and FP) and included five domains. The GA is easily performed even in this context of acute care and only takes a few additional ten minutes. Comorbidities were assessed using the Cumulative Illness Rating Scale for Geriatrics (CIRS(G)) which covers all diseases including hypertension, cardiovascular diseases, diabetes, chronic bronchitis, and their long-term complications (Supplementary Table 1) [15]. Impairment was defined as a total CIRS(G) score above the median of 11. Dependency before hospitalization was defined from a six-item activities of daily living (ADL) score of 5 out of 6 or less, and from a four-item simplified instrumental ADL score (IADL, using the telephone, transport, medications, and money management) of under 4 [16, 17] (Supplementary Table 2). Malnutrition was defined as a body mass index (BMI) under 21 kg/m^2^ or unintentional weight loss in the previous year ≥ 5% [18, 19]. Depressed mood was defined from a Mini-Geriatric Depression Scale score of 1 or more out of 4 (Supplementary Table 3) [20]. Impaired mobility was defined by the presence of muscle weakness (MW) assessed from hand-grip strength. Maximum handgrip strength (in kg) was measured twice for each hand using a hand-held dynamometer (model EH101; Zhongshan Camry Electronic Co., Ltd, Guangdong, China). MW was defined by thresholds adjusted for gender and BMI derived from the frailty phenotype established by Fried et al. [19].

### Covariates

At the time of diagnosis, we collected total lymphocyte count, serum levels of albumin (g/L), C-reactive protein (mg/L), D-Dimers (ng/mL), and Lactate Dehydrogenase (LDH, UI/L). These covariates were expressed as continuous variables. We also tested D-Dimers serum level as an age-adjusted categorical variable according to National consensus (i.e. abnormal D-Dimers ≥ age x 10) [21]. Anticoagulation was classified as follows: curative, preventive or none. Exposure to converting enzyme inhibitors was noted. Exposure to the hydroxychloroquine and azithromycin combined therapy was noted to assess the predictive value for the risk of death with this treatment [22].

### Outcome

Data was collected from 03/28/2020 to 04/13/2020. On 05/02/2020, inpatient mortality following the diagnosis of Covid-19 until discharge from acute care unit was determined. Vital status was obtained from medical records.

### Statistical analyses

Categorical data were expressed as numbers and proportions, and continuous data as means and standard deviation (SD) or medians and interquartile range (IQR).

Comparisons of baseline characteristics between survivors and non-survivors were performed using the log-rank test. A Cox uni- and multivariate proportional-hazard regression model was run to assess factors associated with one-month mortality. Model assumptions were verified. Variables yielding *P* values ≤ 0.25 in the univariate analysis were considered for inclusion in the multivariate analysis using a backward procedure according to the lowest Akaïke Information Criteria. Continuous variables were expressed per one IQR of more. We then assessed interaction terms between predictors. A derivation score for each predictor was created using Hazard Ratio point-based scoring system [23]. We categorized this score by the median. The calibration of the derivation score was assessed by using the Grönnesby and Borgan test. A *P* value ≥ 0.05 was considered to indicate good calibration. Discrimination by the derivation score was assessed using Harrell’s C index with 95%CI. Survival curves were plotted according to the Kaplan-Meier method with the derivation score divided by median. Internal validation was performed with the bootstrap-adjusted Harrell’s C index with 1000 resamples as recommended by the TRIPOD guidelines [24].

All tests were two-sided, and the threshold for statistical significance was set at *P* <0.05. The data was analysed using R statistical software (version 4.0.0, R Foundation for Statistical Computing, Vienna, Austria; http://www.rproject.org).

## Supplementary Material

Supplementary Tables
