# Supplementary material for: ADL-dependency, D-Dimers, LDH and absence of anticoagulation are independently associated with one-month mortality in older inpatients with Covid-19
Source: Aging (Albany NY). 2020 Jun 23;12(12):11306–13. doi: 10.18632/aging.103583 (PMC7343508; doi:10.18632/aging.103583)
Supplement: Supplementary Tables [file aging-12-103583-s001..pdf]

SUPPLEMENTARY TABLES

Supplementary Table 1. The Cumulative Illness Rating Scale for Geriatrics (CIRS(G)) [15].

Disease	Severity				
	0	1	2	3	4
Heart					
Vascular (including hypertension)					
Hematopoietic					
Respiratory					
Eyes, ears, nose, throat, and larynx					
Upper Gastrointestinal					
Lower Gastrointestinal					
Liver, pancreas, and biliary					
Renal					
Genitourinary					
Musculoskeletal and skin					
Neurologic					
Endocrine and breast					
Psychiatric illness					
**Total** (0-56)					

Supplementary Table 2. Score for Activities of Daily Living (ADL) [16].

Questions	Points		
	Does alone 1	Does with help 0.5	Cannot do alone 0
Washing			
Getting dressed			
Moving about indoors			
Going to the toilet			
Eating			
Continence			

Scoring:
Scores range from 0 to 6.
Total score ≤ 5/6 indicates ADL-dependency.

Supplementary Table 3. The mini Geriatric Depression Scale (mini-GDS) [20].

Questions	Points	
	Yes	No
Do you feel discouraged and sad?	1	0
Do you feel your life is empty?	1	0
Are you happy most of the time?	0	1
Do you feel hopeless?	1	0

Scoring:
Score ranges from 0 to 4.
Total score ≥ 1/4 indicates depressed mood.
